# Trifluoperazine inhibits acetaminophen-induced hepatotoxicity and hepatic reactive nitrogen formation in mice and in freshly isolated hepatocytes

**DOI:** 10.1016/j.toxrep.2017.02.005

**Published:** 2017-02-27

**Authors:** Sudip Banerjee, Stepan B. Melnyk, Kimberly J. Krager, Nukhet Aykin-Burns, Sandra S. McCullough, Laura P. James, Jack A. Hinson

**Affiliations:** a Department of Pharmacology and Toxicology, University of Arkansas for Medical Sciences, Little Rock, AR, 72205, United States; b Department of Pediatrics, University of Arkansas for Medical Sciences, and Arkansas Children’s Hospital Research Institute, Little Rock, AR, 72205, United States; c Department of Pharmaceutical Sciences, University of Arkansas for Medical Sciences, Little Rock, AR, 72205, United States

**Keywords:** APAP, Acetaminophen, NAPQI, N-Acetyl-p-benzoquinone imine, GSH, reduced glutathione, APAP-cys, 3-(cystein-S-yl)-acetaminophen, NO, Nitric Oxide, iNOS, Inducible nitric oxide synthase (NOS2), nNOS, Neuronal nitric oxide synthase (NOS1), GSNO, S-Nitrosoglutathione, GSSG, glutathione disulfide, NANT, N-[(4S)-4-amino-5-[(2-aminoethyl) amino] pentyl]-N’-nitroguanidinetris (trifluoroacetate), MPT, Mitochondrial Permeability Transition, LDH, Lactate Dehydrogenase, OCR, Oxygen Consumption Rate, 3-NT, 3-Nitrotyrosine, TFP, Trifluoperazine, Acetaminophen, Neuronal nitric oxide, Oxidative stress, Mitochondria

## Abstract

The hepatotoxicity of acetaminophen (APAP) occurs by initial metabolism to N-acetyl-*p*-benzoquinone imine which depletes GSH and forms APAP-protein adducts. Subsequently, the reactive nitrogen species peroxynitrite is formed from nitric oxide (NO) and superoxide leading to 3-nitrotyrosine in proteins. Toxicity occurs with inhibited mitochondrial function. We previously reported that in hepatocytes the nNOS (NOS1) inhibitor NANT inhibited APAP toxicity, reactive nitrogen and oxygen species formation, and mitochondrial dysfunction. In this work we examined the effect of trifluoperazine (TFP), a calmodulin antagonist that inhibits calcium induced nNOS activation, on APAP hepatotoxicity and reactive nitrogen formation in murine hepatocytes and *in vivo*. In freshly isolated hepatocytes TFP inhibited APAP induced toxicity, reactive nitrogen formation (NO, GSNO, and 3-nitrotyrosine in protein), reactive oxygen formation (superoxide), loss of mitochondrial membrane potential, decreased ATP production, decreased oxygen consumption rate, and increased NADH accumulation. TFP did not alter APAP induced GSH depletion in the hepatocytes or the formation of APAP protein adducts which indicated that reactive metabolite formation was not inhibited. Since we previously reported that TFP inhibits the hepatotoxicity of APAP in mice without altering hepatic APAP-protein adduct formation, we examined the APAP treated mouse livers for evidence of reactive nitrogen formation. 3-Nitrotyrosine in hepatic proteins and GSNO were significantly increased in APAP treated mouse livers and decreased in the livers of mice treated with APAP plus TFP. These data are consistent with a hypothesis that APAP hepatotoxicity occurs with altered calcium metabolism, activation of nNOS leading to increased reactive nitrogen formation, and mitochondrial dysfunction.

## Highlights

• Increased reactive nitrogen and oxygen species formation leads to APAP hepatoxicity.
• TFP is known to block nNOS both *in vivo* as well as in vitro.
• The nNOS inhibitor TFP blocks toxicity and the increased RNS/ROS formation.
• Toxicity occurs with increased 3- nitro tyrosine both in vivo as well as in *vitro*.
• NNOS inhibition by TFP leads to decreasing 3-nitro tyrosine *in vivo* as well as *in vitro*.

## Graphical abstract

**Figure:**
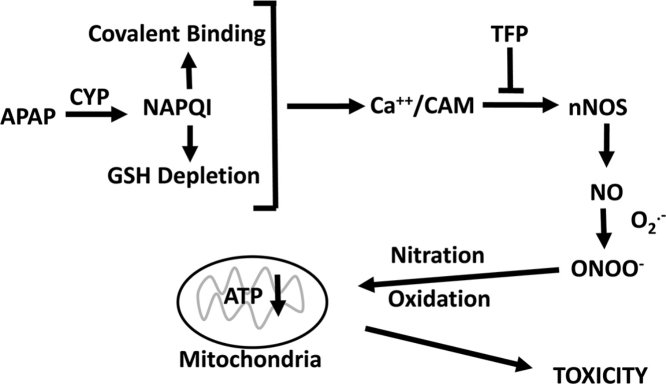

## 1 Introduction

One of the most widely used analgesic/antipyretic drug in the world is acetaminophen (paracetamol, APAP; N-acetyl-*p*-aminophenol). At therapeutic doses APAP is believed to be safe but when an overdose it may cause hepatic centrilobular necrosis [1], [2]. Annually approximately 500 deaths occur in the United States due to overdose of APAP [3]. Because of their susceptibility to acute doses of APAP and the similarity to the human toxicity, mice have been the most frequently studied experimental model for APAP toxicity studies. The deduced mechanisms have proven to be applicable to the human toxicity. These studies have revealed that, APAP is metabolized in hepatocytes by cytochrome P450 (CYP) to the reactive metabolite N-acetyl-*p*-benzoquinone imine (NAPQI) [4]. Following a therapeutic dose, glutathione (GSH) efficiently detoxifies NAPQI but in overdose total hepatic GSH is depleted [2], [5]. Under conditions of GSH depletion NAPQI reacts with available sulfhydryl on proteins to form 3-(cystein-S-yl)-acetaminophen (APAP-Cys) protein adducts [6]. This so called covalent binding correlates with hepatic toxicity. The type of toxicity is hepatocyte necrosis.

Whereas the exact mechanism by which necrosis occurs is unclear, available data suggest that oxidant stress mediated by reactive nitrogen may play a significant role. Previously our laboratory reported the presence of nitrated tyrosine (3-nitrotyrosine) in hepatic proteins of APAP-treated mice [7]. The nitrated proteins occurred in the same hepatocytes that contained APAP-protein adducts and were undergoing necrotic changes. Nitration of tyrosine was postulated to be mediated by peroxynitrite, a highly reactive species generated from superoxide and nitric oxide (NO) [8], [9], [10]. Peroxynitrite is both a nitrating as well as an oxidizing agent and can be detoxified by GSH [11]. GSH is depleted in APAP toxicity [2]. Our laboratory reported nitration of MnSOD with loss of activity [12]. In addition, mitochondrial aldehyde dehydrogenase, glutathione peroxidase, ATP synthase, and 3-ketoacyl-CoA thiolase [13] have been reported to be nitrated in murine APAP toxicity. N-Acetylcysteine (NAC) treatment has been reported to decrease hepatic protein nitration in APAP toxicity [13].

A major focus of our research has been to understand the role of reactive nitrogen in APAP hepatotoxicity. We and others have previously shown that iNOS (inducible nitric oxide synthase) knockout mice are equally sensitive to APAP hepatotoxicity as wild type mice [14], [15] and we reported that pharmacological inhibitors of iNOS do not decrease hepatotoxicity in mice [16]. ubsequently we examined APAP in freshly isolated hepatocytes. These hepatocytes still have high levels of CYP enzymes necessary for metabolism of APAP to the reactive metabolite NAPQI leading to GSH depletion and covalent binding. Two pharmacological inhibitors of iNOS (L-NIL and SAIT) did not decrease toxicity; however, the neuronal nitric oxide synthase (nNOS) inhibitor 7-nitroindazole inhibited toxicity [17]. We reported that hepatotoxicity of APAP in mice was delayed in nNOSα knockout mice compared to the wild type mice [18]. Moreover, we recently reported that the specific nNOS inhibitor NANT inhibited APAP toxicity in freshly isolated hepatocytes and blocked production of reactive nitrogen and oxygen species [19]. These data led to the hypothesis that nNOS is the source of the NO leading to oxidant stress in APAP hepatotoxicity. nNOS is known to occur in hepatocytes [20].

Trifluoperazine (TFP) has been previously reported to block APAP toxicity in mice [21], [22], [23]. Our laboratory previously examined APAP toxicity in freshly isolated hepatocytes, a model of toxicity where GSH levels and CYP levels are still high compared to cultured hepatocytes and time to toxicity is similar to that occurring in vivo in mice. We found that TFP inhibited APAP toxicity in freshly isolated hepatocytes [17]. Since TFP is a mitochondrial permeability transition (MPT) inhibitor and another MPT inhibitor, cyclosporine A, blocked APAP toxicity [24], [25], MPT was postulated to be important in toxicity. However, the molecular events causing the APAP-induced MPT are poorly defined. To further understand the mechanism of how TFP blocked APAP toxicity, Chaudhuri et al. [21], examined the effect of APAP on hepatic phospholipase A2 (PLA2) activity. Mehendale and coworkers [26] previously postulated a role of phospholipase A2 in APAP induced liver injury. Histological evidence of the APAP induced necrosis and APAP-induced increases of serum ALT were significantly decreased by co-administration with TFP: however, APAP induced GSH depletion and covalent binding were not significantly altered by co-administration with TFP. In livers of APAP treated mice there was a significant early increase in both cytosolic and secretory PLA2 activities, both were partially decreased by co-administration with TFP but the role of PLA2 in APAP toxicity was unclear [21]. However, TFP inhibits the calcium-calmodulin activation of nNOS activity by inhibiting electron flow [27], [28], [29]. Therefore, in this manuscript we have examined the effect of TFP on APAP toxicity and reactive nitrogen and oxygen formation in freshly isolated hepatocytes, and hepatic reactive nitrogen formation in APAP treated mice.

## 2 Materials and methods

### 2.1 Reagents

Acetaminophen (APAP; 4-acetamidophenol), Percoll, Hepes, (heparin sodium salt grade I-A from porcine intestinal mucosa), penicillin G (sodium salt), RPMI-1640 modified media (with L-glutamine and without sodium bicarbonate and phenol red), 0.4% trypan blue solution, GSH, GSSG, *S*-nitrosoglutathione (GSNO), 3NT, NADH, NAD, and trifluoperazine (TFP; 10-[3-(4-methylpiperazin-1-yl)propyl]-2-(trifluoromethyl)-10*H*-phenothiazine), ATP Bioluminescent Assay kit, were products of Sigma Chemical Company (St Louis, MO). Collagenase A from Clostridium histolyticum was purchased from Roche Diagnostics (Indianapolis, IN). MitoSOX Red, 4-Amino-5-methylamino-2′,7′-difluorofluorescein diacetate (DAF-FM), and JC1 were obtained from Life technologies (Eugene, OR). LDH (lactate dehydrogenase) cytotoxicity detection kit was a product of from Roche Diagnostic Corporation (Indianapolis).

### 2.2 Animals

Male 6-week old mice (B6C3F1) were bred and pruchase from Harlan Laboratories (Indianapolis, IN). All animal experimentation and protocols was approved by the UAMS Institutional Animal Care and Use Committee (IACUC). Experiments were carried out in accordance with the Guide for the Care and Use of Laboratory Animals as published by the U.S. National Institutes of Health. Mice were fed *ad libitum* and were acclimated one week prior to sacrifice.

### 2.3 Hepatocyte isolation and incubation mixtures

As previously described freshly isolated hepatocytes were obtained from mice by collagenase perfusion [17], [19] Hepatocytes >40 million cell viability >90% was determined by Trypan blue exclusion. The hepatocytes (1 million cells/ml) in RPMI 1640 media supplemented with 25 mM HEPES, 10 IU heparin/ml, and 500 IU penicillin G/ml were incubated in 125 ml Erlenmeyer flasks at 37 °C under an atmosphere of 95% O_2_-5% CO_2_. APAP (1 mM) was added to experimental hepatocytes [17]. Control flasks did not contain APAP. Some incubation contained 10 μM TFP. Incubations were performed in three or four separate experiments performed on different days.

### 2.4 Spectrophotometric assays

Toxicity was determined by LDH release from hepatocytes as previously reported [19]. Briefly, the hepatocytes were isolated from the media by centrifugation. Following kit directions the supernatants (100 μl) was mixed with the reaction mixture from the detection kit (100 μl) and subsequently heated at 37 °C for 30 min in the dark. The absorbance of the samples was determined spectrophotometrically in a Bio-rad 550 plate reader at a 490 nm. Cytotoxicity was evaluated as previously described [19]. reactive oxygen (superoxide) was evaluated by increased fluorescence of MitoSOX Red as previously described [30], [31]. 4-Amino-5-methylamino-2′,7′-difluorofluorescein diacetate (DAF- FM) was utilized for assay of reactive nitrogen (NO) [32]. Briefly, hepatocytes (1 ml) were centrifuged at 140 × *g* for 2 min and supernatant discarded. The hepatocytes were resuspended with in 2 ml of phosphate-buffered saline containing DAF-FM (10 μM) or MitoSOX (5 μM) and incubated at 37oC for 20 min in an atmosphere of 95% O2/5% CO2. The cells were subsequently centrifuged to remove excess dye and resuspended in 2 ml of phosphate-buffered saline. Fluometrical analysis for Mitosox for DAF-FM (excited/emitted at 495/515 nm) and for MitoSOX (510/580 nm) was performed using a SpectraMax M2e fluorescence spectrophotometer. The relative mitochondrial membrane potential was determined using JC1, a mitochondrial membrane specific cationic as previously described [19]. Briefly, hepatocytes (2 ml) were centrifuged at 140*g* for 2 min and the supernatant discarded. Cells were resuspended in 3 ml JC1 buffer (6.5 μM JC1) and incubated for 25 min at 37 °C in atmosphere of 95% O_2_/5% CO_2_. Subsequently, cells were centrifuged and washed to remove excess dye and resuspended in JC1 buffer (2 ml). Fluometric analysis was performed by excitation at 490 nm and emission at 530 and 590 nm. The ratio of absorbance at 590 nm to 530 nm (590:530 ratio) was evaluated as the relative mitochondrial membrane potential [25], [33]. Adenosine 5′-triphosphate (ATP) production in isolated hepatocytes was determined utilizing manufacturer’s protocol, using a TD20/20 luminometer (Turner Design, Sunnyvale, CA, USA). Quantification of ATP was obtained from an ATP standard curve. These assays described were performed as we previously reported [19].

### 2.5 HPLC assays

High-performance liquid chromatography (HPLC) was used to quantify GSH, GSSG, GSNO and 3-nitrotyrosine (3-NT). Briefly, approximately 2 million hepatocytes were homogenized in ice-cold phosphate-buffered saline (PBS) buffer and protein s were precipitated by treatment with 10% metaphosphoric acid for 30 min on ice. The samples were centrifuged at 18,000*g* at 4 °C for 15 min. Twenty μl of the resulting supernatants were injected into the HPLC column for metabolite quantification. The pellet was used for protein analysis using BCA protein assay. The methodological details for HPLC analysis of GSH, GSSG, GSNO and 3-NT have been previously described [34], [35]. NAD^+^ and NADH levels were determined utilizing a Dionex Ultimate 3000 HPLC-UV system as previously described [36]. APAP covalently bound to proteins (APAP-cysteine) in hepatocytes was determined following protease treatment of hepatocyte homogenates followed by high performance liquid chromatography-electrochemical analysis for as previously reported [37].

### 2.6 Oxygen consumption rate

Utilizing previously described methods the Oxygen consumption rate (OCR) was determined at 37 °C using an XF96 extracellular flux analyzer (Seahorse Bioscience, Billerica, MA) [19]. Briefly, freshly isolated hepatoyctes (8000 per well) were plated in CellTak coated plates, using unbuffered DMEM containing 4 mM glutamate and incubated in a non-CO_2_ incubator at 37 °C for 1 h. Baseline measurements (three) were acquired before sequential injection of TFP (10 μM) followed by APAP (1 mM). Oxygen consumption rates were calculated using the Seahorse XF-96 software. The data represent an average of 20–32 measurements on two different days (10–16 wells per mouse per day).

### 2.7 Statistical analyses

Analysis of variance was performed with a Bonferroni *post hoc* test using the Prism GraphPad 6.0 (San Diego, CA). Statistical significance was defined as the experimental being *p <* 0.05 compared to control.

### 2.8 APAP toxicity, GSH depletion, and protein adduct formation in hepatocytes

**Fig 1:**
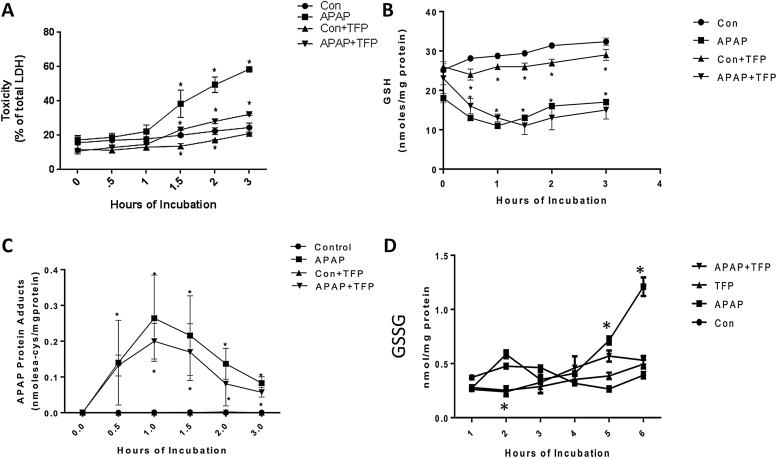
Effect of TFP on APAP-induced toxicity, glutathione, and APAP-CYS in freshly isolated hepatocytes. Hepatocytes were incubated with APAP (1 mM), APAP plus TFP (10 uM), TFP alone or with media alone (Con) for 0–3 h. (**A**) relative toxicity (LDH release), (**B**) GSH, (**C**) APAP-CYS, and (**D**) GSSG in hepatocytes. Significant increase was indicated by * (p ≤ 0.05) from control group. Samples were n = 3 from 3 separate mice and hepatocytes were isolated on 3 different days. The data are presented as mean ± SE.

The effect of TFP on APAP mediated toxicity in freshly isolated hepatocytes (hepatocyte incubations) was investigated to determine the role of nNOS. TFP has been reported to be an inhibitor of nNOS [29]. Toxicity (LDH release into media) was significantly increased in APAP treated hepatocytes (Fig. 1A) at 1.5–3 h compared to control hepatocytes. Addition of 10 μM TFP eliminated toxicity produced by APAP (Fig. 1A).

APAP metabolism by CYP enzymes to the reactive metabolite NAPQI and its role in GSH depletion and covalent binding have been described [1]. Since inhibition of CYP mediated APAP metabolic activation is known to decrease toxicity, the effect of TFP on GSH depletion and covalent binding by APAP was evaluated. In the APAP treated hepatocytes GSH levels were significantly depleted by 70% at 0.5 h compared to the control hepatocytes (Fig. 1B). APAP mediated GSH depletion was not altered by TFP. Interestingly, oxidized GSH (GSSG) levels were significantly increased to APAP alone compared to the APAP/TFP treated hepatocytes; however, the amount is low compared to total GSH (Fig. 1D). These data are consistent with some oxidative stress occurring in the APAP only treated hepatocytes [38]. As shown in Fig. 1C, TFP did not alter covalent binding of APAP to protein (APAP cysteine protein adducts). These data and the GSH depletion data, indicate that the mechanism by which TFP decreased APAP toxicity in hepatocytes was not by inhibition of CYP metabolism of APAP to form NAPQI leading to GSH depletion and APAP covalent binding (Cys adduct formation).

### 2.9 Formation of reactive oxygen and nitrogen species in hepatocytes

**Fig. 2:**
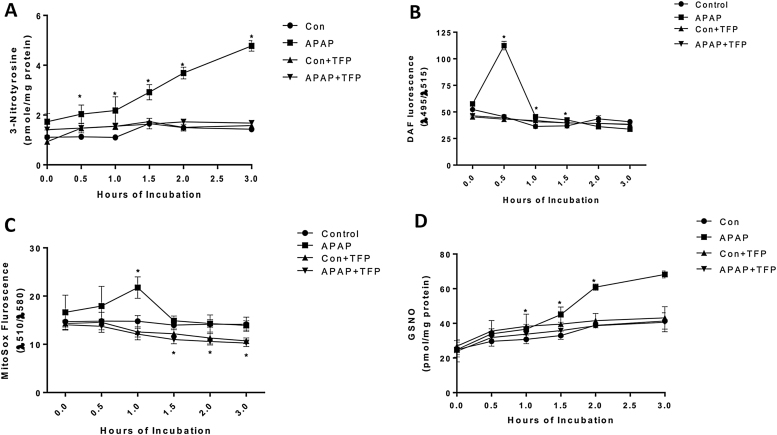
Effect of TFP on APAP-induced reactive nitrogen and oxygen formation in freshly isolated hepatocytes. Hepatocytes were incubated with APAP (1 mM), APAP plus TFP (10uM), TFP alone or with media alone (Con) for 0–3 h. (**A**) 3-NT in proteins, **(B)** Nitric Oxide **(C**) Superoxide production, and (**D)** GSNO in hepatocytes. Significant increase was indicated by * (p ≤ 0.05) from control group. Samples were n = 3 from 3 separate mice and hepatocytes were isolated on 3 different days. The data are presented as mean ±SE.

Since TFP has been reported to be a nNOS inhibitor its effect on reactive nitrogen formation in APAP toxicity was determined. 3-Nitrotyrosine in hepatocyte proteins of the APAP treated mice was quantified. 3-Nitrotyrosine is believed to be formed by nitration of tyrosine residues in proteins by peroxynitrite, a reactive nitrogen species formed from NO and superoxide. 3-Nitrotyrosine levels in proteins were significantly increased at 0.5 h in APAP treated hepatocytes compared to control hepatocytes and remained significantly increased to 3.0 h (Fig. 2A). 3-Nitrotyrosine levels remained at control levels for the duration of the experiment (0–3 h) (Fig. 2A).

Since peroxynitrite is formed from nitric oxide (NO) and superoxide the effect of TFP on these reactive species was determined. NO levels in APAP treated hepatocytes significantly increased at 0.5 h and subsequently decreased. In hepatocytes containing APAP plus TFP, NO levels were not significantly different from control levels at 0–3 h (Fig. 2B). In APAP treated hepatocytes superoxide levels were significantly increased at 1 h and subsequently decreased to control levels. TFP blocked the increase in superoxide at 1 h. Interestingly, in the presence of TFP with or without APAP, background levels of superoxide were significantly inhibited (1.5–3 h) compared to non-TFP treated hepatocytes.

The formation of GSNO was also quantified in the hepatocytes. Nitrosylation of proteins has been postulated to important in cell signaling [39]. GSNO was significantly increased in the APAP treated hepatocytes at 1.5–3 h (Fig. 2D). TFP significantly blocked the APAP induced increase in GSNO formation.

### 2.10 Alteration in NAD^+^ and NADH levels in hepatocytes

**Fig. 3:**
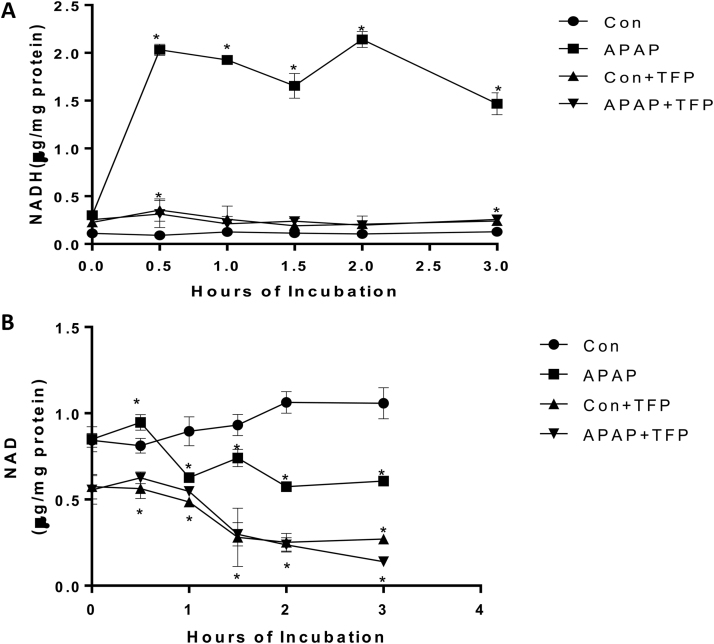
Effect of APAP and TFP on NAD and NADH levels in freshly isolated hepatocytes. Hepatocytes were incubated with APAP (1 mM), APAP plus TFP (10 uM), TFP alone and with media alone (Con) for 0–3 h. (**A**) NADH and (**B**) NAD + in the hepatocytes. Significant increase was indicated by * (p ≤ 0.05) from control group. Samples were n = 3 from 3 separate mice and hepatocytes were isolated on 3 different days. The data are presented as mean ± SE.

Hepatocyte treated with APAP were found to have a dramatic increase in NADH levels starting at 0.5 h and continuing the duration of the incubation time compared to control hepatocytes. TFP blocked the APAP induced increase in NADH levels in the hepatocytes. NAD levels were significantly lower in APAP, APAP plus TFP, and TFP hepatocytes compared to control hepatocytes at 3 h. (Fig. 3B).

### 2.11 Oxygen Consumption Rate (OCR), mitochondrial membrane potential and ATP production in hepatocytes

**Fig. 4:**
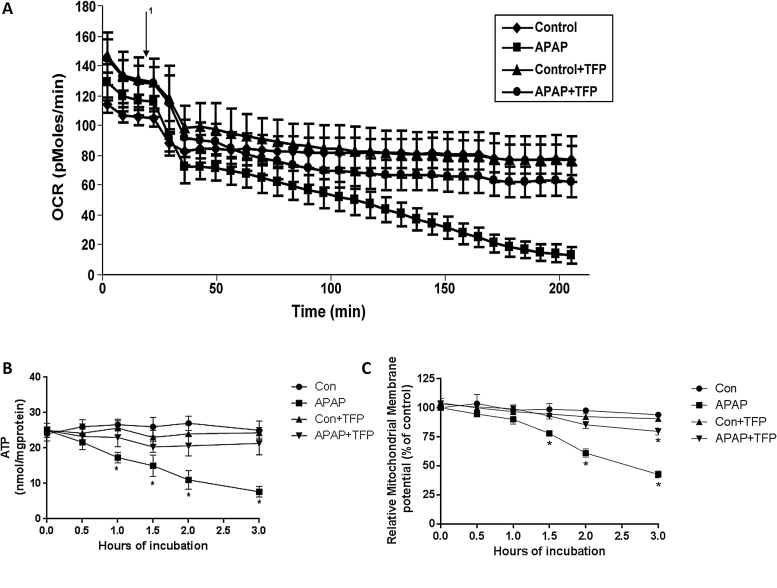
Effect of TFP on APAP-induced alterations of oxygen consumption, mitochondrial membrane potential, and ATP levels in freshly isolated hepatocytes. Hepatocytes were incubated with APAP (1 mM), APAP plusTFP (10 uM), TFP alone and with media alone (Con) for 0–3 h. Oxygen Consumption Rate (OCR) (**A**) in the above mention groups were measured using Seahorse XF96 analyzer 1 in the graph represent the time at which APAP is injected in the groups and TFP in the groups. (**B)** Relative mitochondrial membrane potential and (**C**) ATP production in the hepatocytes. Significant increase was indicated by * (p ≤ 0.05) from control group. Samples were n = 3 from 3 separate mice and hepatocytes were isolated on 3 different days. The data are presented as mean ± SE.

Using an extracellular flux analyzer the effect of APAP on OCR was determined. A decrease in OCR was observed between 0 and 0.5 h in both APAP treated hepatocytes and in control hepatocytes. Subsequently, there was a gradual decrease in OCR between 1 and 3 h in the APAP treated hepatocytes (Fig. 4A). TFP blocked this decrease in the decreased OCR in the APAP treated hepatocytes.

In previous research we reported that APAP caused a loss of mitochondrial membrane potential [19], [25]. The effect of TFP on APAP induced alterations in mitochondrial membrane potential in the hepatocytes was thus examined. Mitochondrial membrane potential was significantly reduced in hepatocytes treated with APAP between 1.5–3 h (Fig. 4B) compared to control hepatocytes. TFP inhibited the APAP induced loss of mitochondrial membrane potential. The effect of APAP on ATP levels in the hepatocytes was also examined. ATP levels in APAP treated hepatocytes were significantly decreased from 0.5 to 3 h (Fig. 4C), compared to control hepatocytes. Inclusion of TFP in the APAP treated hepatocytes blocked the decrease in ATP production.

### 2.12 APAP hepatotoxicity and reactive nitrogen formation in livers of mice

**Fig. 5:**
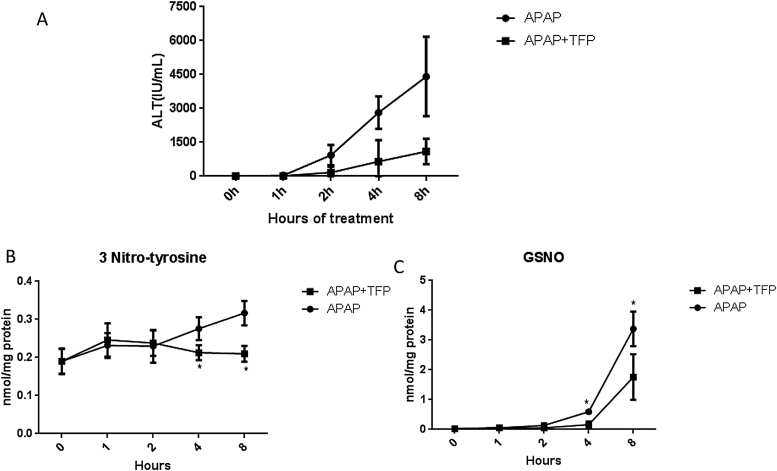
Effect of TFP on APAP hepatotoxicity and reactive nitrogen formation in mice. Mice were administered APAP (200 mg/kg) or APAP plus TFP (10 mg/kg). TFP was administered 1 h before APAP. Serum and livers samples were collected at the indicated times. **(A)** Serum ALT data were previously published as a bar graph (Chaudhuri et al., 2012) and the data have been replotted as a line graph. The livers were frozen at −80° C until analysis. **(B)** 3-Nitrotyrosine protein adducts. **(C)** Nitrosoglutathione (GSNO) in hepatocytes.

Previously we reported that APAP (200 mg/kg) increased hepatic necrosis and ALT levels at 4, 8, 24 and 48 h [21]. TFP (10 mg/kg) administered 1 h prior to APAP significantly decreased the resulting histological necrosis and ALT levels at 4, 8, 24, and 48 h, without altering APAP hepatic covalent binding. Data from our previous experiment have been replotted in a line graph and are provided in Fig. 5A. The livers have now been analyzed for evidence of reactive nitrogen formation (3-nitrotyrosine in hepatic proteins and hepatic GS-NO). 3-Nitrotyrosine in hepatic protein (Fig. 5B) and hepatic GSNO levels (Fig. 5C) were significantly increased in the APAP treated mice and were significantly decreased in the livers of the APAP plus TFP treated mice.

## 3 Discussion

In previous research we reported TFP decreased APAP toxicity in hepatocytes [25] nd in mice without altering metabolic activation [21]. TFP has been previously reported to be a MPT inhibitor and MPT had been implicated in APAP toxicity [25]. Since Mehendale and coworkers [26] postulated a role for phospholipase A2 (PLA2) in progression of liver injury induced by APAP toxicity we previously examined its role in APAP hepatotoxicity and the effect of TFP. In mice we found that cytoplasmic and secretary phospholipase A2 (sPLA2) were increased in murine APAP hepatotoxicity. The levels of these enzymes were significantly decreased in mice treated with TFP plus APAP.

Since TFP has been previously reported to be a nNOS inhibitor, in this manuscript we examined its effect on APAP induced reactive nitrogen and oxygen species formation. We previously reported that two nNOS inhibitors (7-nitroindazole and NANT) blocked APAP toxicity in freshly isolated hepatocytes. Freshly isolated hepatocytes are superior to cultured hepatocytes because CYP levels and GSH levels are similar to those *in vivo* whereas in cultured hepatocytes CYP levels are very low. The nNOS inhibitor 7-nitroindazole was found to inhibit APAP mediated toxicity, 3-nitrotyrosine formation, and loss of mitochondrial membrane potential when added in the late phase of toxicity [17]. More recently, we examined the effect of the nNOS inhibitor NANT. Importantly, NANT did not inhibit metabolic activation of APAP and was used to examine the effect of nNOS on early events in APAP toxicity in hepatocytes. It was found that NANT when added at time 0 inhibited APAP mediated toxicity, and APAP mediated formation of reactive nitrogen and oxygen species (NO, 3-nitrotyrosine in protein, superoxide, and GSNO). APAP caused a decrease in the hepatocyte oxygen consumption rate, loss of mitochondrial membrane potential, and a decrease in ATP production. All of the events were blocked by NANT [19]. In addition, APAP also caused a dramatic increase in NADH which was blocked by NANT. This latter finding suggested that APAP toxicity occurred with inhibition of mitochondrial complex I. Three possibilities were suggested whereby reactive nitrogen species could produce mitochondrial dysfunction leading to toxicity: 1) peroxynitrite mediated nitration of critical mitochondrial proteins in Complex I, 2) peroxynitrite mediated oxidation of ubiquinol, or 3) GSNO mediated nitrosylation of critical mitochondrial proteins in Complex I [19]. In this manuscript we have extended our investigation on the role of nNOS and reactive nitrogen in APAP hepatotoxicity by examining the effect of TFP on APAP mediated toxicity and reactive nitrogen and oxygen formation both in freshly isolated hepatocytes and in mice. Previously we reported that TFP blocked APAP toxicity in freshly isolated hepatocytes when added in the late phase of toxicity [25]. In this manuscript we found that addition of TFP at time 0 to the APAP containing hepatocyte incubations inhibited APAP toxicity (Fig. 1), protein nitration (Fig. 2A), NO formation (Fig. 2B), reactive oxygen formation (superoxide) (Fig. 2C), and GSNO formation (Fig. 2D). Toxicity and reactive nitrogen and oxygen species formation were accompanied by decreased oxygen consumption rate (Fig. 4A), decreased ATP production (Fig. 4B), and decreased mitochondrial membrane potential (Fig. 4C). All of these decreases were significantly blocked in the incubations containing APAP plus TFP. In addition, we report that APAP induced accumulation of NADH, loss of mitochondrial membrane potential, and inhibition of ATP biosynthesis are reversed when TFP is included in the APAP containing incubations. Thus, TFP is an effective inhibitor of APAP toxicity and formation of reactive nitrogen and oxygen species. These data are similar to what we previously reported for the nNOS inhibitor NANT and clearly indicate a role for reactive nitrogen and oxygen species in APAP hepatotoxicity.

Importantly, TFP blocks APAP induced hepatic necrosis and increased serum ALT levels in mice Chaudhuri et al. [21]. These ALT data, which are replotted as a line graph in Fig. 5A, shows that APAP alone increased serum ALT to approximately 4500 IU/L by 8 h. Co-administration of TFP (10 mg/kg) with the APAP decreased ALT at this time to approximately 1000 IU/L. Higher doses of TFP were not examined. Thus, it is unclear if higher doses would have completely eliminated toxicity [21]. However, the major point of this experiment is that TFP decreases APAP toxicity. It is unclear if TFP has any clinical application to APAP toxicity in humans.

The finding that TFP blocks APAP hepatotoxicity suggests altered calcium metabolism is a key event leading to toxicity. In response to elevated calcium nNOS binds to calmodulin leading to synthesis of NO [27], [28]. TFP blocks activation of nNOS by calcium/calmodulin and thus inhibits NO formation. The mechanism appears to be a distortion of the calmodulin structure necessary for activation [29]. Interestingly, PLA2 activity is also mediated by a calcium/calmodulin activation and thus may explain our previous finding that PLA2 activity is increased in APAP hepatotoxicity [21].

Altered calcium metabolism has been previously postulated to be important in APAP hepatotoxicity [40], [41], [42], [43]. Burcham and Harmon [41] and Timerstein and Nelson [43] reported increases in hepatic calcium levels following toxic doses of APAP to mice. Tsokos-Kuhn and coworkers reported that toxic doses of APAP significantly decreased hepatic plasma membrane calcium-ATPase activity [43], [44]. This enzyme is important in the removal of calcium from the cell because extracellular calcium enters the cell due to a large electrochemical gradient driving this ion into the cell [45]. Inhibition of the enzyme results in calcium accumulation in the cell. The authors suggested that covalent binding of the reactive metabolite of APAP to the calcium-ATPase was responsible for the loss of its activity [43], [44]. This enzyme has not been reported to be adducted by the reactive metabolite NAPQI; however, high levels of protein adducts were observed in the hepatic plasma membrane fraction of APAP treated mice [46].

The effect of the calcium specific chelators on APAP induced toxicity in freshly isolated hamster hepatocytes was examined by Boobis et al. [47]. The calcium chelator Quin 2-AM prevented the loss of viability [47]. Moreover, they found that an increase in cytosolic calcium correlated with development of toxicity [48]. Corcoran et al. found a toxic dose APAP to mice caused an increase in nuclear calcium levels using cultured hepatocytes [49] and that EGTA, a calcium ion chelator, inhibited hepatocyte death; a finding that suggested toxicity was mediated by extracellular calcium since EGTA is ionized and does not enter the cells. In freshly isolated hepatocytes we previously found that EGTA, as well as Quin-2, inhibited APAP hepatotoxicity [50].

It has been recently reported that the transient receptor potential melanostatine 2 (TRPM2) channels are increased in APAP toxicity [51]. TRPM2 channels are calcium-permeable cation channels that are regulated by free intracellular ADP-ribose and are activated in response to oxidative stress induced by hydrogen peroxide. It was shown that preincubation of hepatocytes with APAP or hydrogen peroxide and subsequent addition of calcium to the media resulted in increased levels of calcium in the cells. The increases were blocked by addition of TRPM2 siRNA but not by control siRNA. TRPM2 knockout mice were shown to be significantly less sensitive to APAP hepatotoxicity (increases in ALT, AST and cellular necrosis) compared to wildtype mice. However, GSH depletion and covalent binding were not analyzed to be certain that differences in APAP metabolic activation did not occur.

**Fig. 6:**
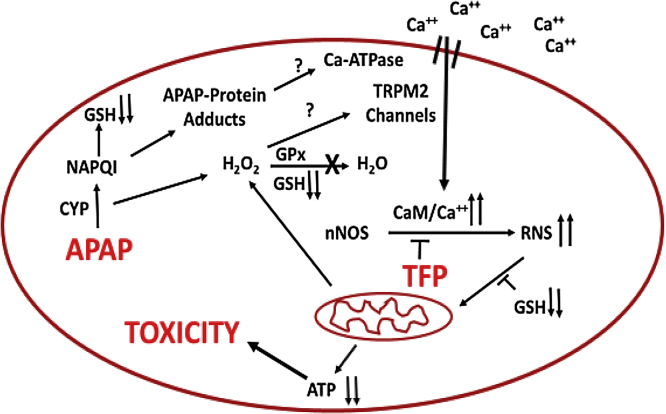
Postulated mechanisms important in APAP mediated toxicity.

A role for TRPM2 channels is particularly intriguing because previous data have suggested a role for Fenton mediated oxidative stress in APAP hepatotoxicity. Hydrogen peroxide has been reported to accumulate in livers of APAP treated mice before development of toxicity [52]. This increase correlates with the depletion of GSH which is important in its detoxification. Hydrogen peroxide plus ferrous ions catalyze a Fenton mediated oxidative stress reaction and the iron chelator deferoxamine (DFO) has been reported to decrease the toxicity of acetaminophen in both rat and mouse hepatocytes [50], [53], [54]. Moreover, administration of DFO to either rats [55] or mice [56] significantly delayed development of toxicity. Lemasters and coworkers reported that in APAP hepatocyte toxicity there is an early increase in cytosolic ferrous ions [57]. Thus in APAP toxicity a Fenton mediated oxidative stress (ferrous ions plus hydrogen peroxide) may lead to activation of TRPM2 channels resulting in increased levels of calcium in the hepatocyte. Activation of TMR2 channels results in extracellular calcium entering the hepatocyte with activation of nNOS via a calmodulin mediated mechanism (Fig. 6). Thus, APAP hepatotoxicity may involve not only a Fenton mediated oxidative stress but as shown in this manuscript a reactive nitrogen mediated oxidative stress.

In this manuscript we show that APAP hepatotoxicity both in mice and in hepatocyte can be decreased by the calmodulin inhibitor trifluoperazine with a decrease in reactive nitrogen formation. The data strong suggest that critical steps in APAP hepatotoxicity are: alteration of intracellular calcium, activation of nNOS leading to formation of reactive nitrogen, and mitochondrial dysfunction with decreased ATP production. These steps are summarized in Fig. 6.

## Funding

This work was supported by the National Institutes of Health, National Institute of Diabetes and Digestive and Kidney Diseases to JAH [Grant R01-DK75936]. A part of the work was supported by National Institute of Environmental Health Sciences [Grant R15 ES022781], National Institute of General Medical Sciences [Grant P20 GM109005] and Arkansas Science and Technology Authority [ASTA 15-B-19] to NAB and KJK. A part of the work was supported by Arkansas Biosciences Institute (ABI) Grant to SBM.

This work was has not been previously presented.

## Authorship contributions

Participated in research design: Banerjee, Melnyk, Aykin-Burn, James, Hinson.

Conducted experiments: Banerjee, Melnyk, Krager, Aykin-Burn, McCullough.

Contributed new reagents or analytic tools: Melnyk, Krager, Aykin-Burn, James.

Performed data analysis: Banerjee, Melnyk, Aykin-Burn, James, Hinson.

Wrote or contributed to the writing of the manuscript: Banerjee, Melnyk, Aykin-burn, James, Hinson.
